# Microbial assemblage and palaeoenvironmental reconstruction of the 1.38 Ga Velkerri Formation, McArthur Basin, northern Australia

**DOI:** 10.1111/gbi.12331

**Published:** 2019-02-07

**Authors:** Amber J. M. Jarrett, Grant M. Cox, Jochen J. Brocks, Emmanuelle Grosjean, Chris J. Boreham, Dianne S. Edwards

**Affiliations:** ^1^ Geoscience Australia Canberra Australian Capital Territory Australia; ^2^ Department of Earth Sciences Centre for Tectonics Resources and Exploration (TRaX) The University of Adelaide Adelaide South Australia Australia; ^3^ Research School of Earth Sciences Australian National University Acton Australian Capital Territory Australia

**Keywords:** anoxia, biomarkers, eukaryotes, euxinia, hydrocarbons, McArthur Basin, phototrophic sulphur bacteria, Proterozoic, redox

## Abstract

The *ca*. 1.38 billion years (Ga) old Roper Group of the McArthur Basin, northern Australia, is one of the most extensive Proterozoic hydrocarbon‐bearing units. Organic‐rich black siltstones from the Velkerri Formation were deposited in a deep‐water sequence and were analysed to determine their organic geochemical (biomarker) signatures, which were used to interpret the microbial diversity and palaeoenvironment of the Roper Seaway. The indigenous hydrocarbon biomarker assemblages describe a water column dominated by bacteria with large‐scale heterotrophic reworking of the organic matter in the water column or bottom sediment. Possible evidence for microbial reworking includes a large unresolved complex mixture (UCM), high ratios of mid‐chained and terminally branched monomethyl alkanes relative to *n*‐alkanes—features characteristic of indigenous Proterozoic bitumen. Steranes, biomarkers for single‐celled and multicellular eukaryotes, were below detection limits in all extracts analysed, despite eukaryotic microfossils having been previously identified in the Roper Group, albeit largely in organically lean shallower water facies. These data suggest that eukaryotes, while present in the Roper Seaway, were ecologically restricted and contributed little to export production. The 2,3,4‐ and 2,3,6‐trimethyl aryl isoprenoids (TMAI) were absent or in very low concentration in the Velkerri Formation. The low abundance is primary and not caused by thermal destruction. The combination of increased dibenzothiophene in the Amungee Member of the Velkerri Formation and trace metal redox geochemistry suggests that degradation of carotenoids occurred during intermittent oxygen exposure at the sediment–water interface and/or the water column was rarely euxinic in the photic zone and likely only transiently euxinic at depth. A comparison of this work with recently published biomarker and trace elemental studies from other mid‐Proterozoic basins demonstrates that microbial environments, water column geochemistry and basin redox were heterogeneous.

## 1 INTRODUCTION

The relationship between ocean chemistry, microbial metabolisms, and the evolution and proliferation of eukaryotes are themes of long‐standing interest in Proterozoic geobiology (Anbar & Knoll, 2002; Johnston et al., 2012; Nursall, 1959). The Mesoproterozoic (1.6–1.0 Ga) Earth is characterised by persistently low atmospheric *p*O_2_ (Planavsky et al., 2014; Scott et al., 2008; Zhang et al., 2016), pervasively oxygen‐depleted oceans (Canfield, 2004; Frei, Gaucher, Poulton, & Canfield, 2009; Lyons & Reinhard, 2009; Poulton, Fralick, & Canfield, 2004) and an apparently stable carbon cycle, as recorded by the carbon isotope ratios of marine carbonates (δ^13^C_carb_: Kaufman, 1997). Consequently, the Mesoproterozoic has generally been considered a period of palaeoenvironmental stability within the Earth system, comprising the core of the so‐called “boring billion” years (1.8–0.8 Ga; Brasier & Lindsay, 1998; Buick, Des Marais, & Knoll, 1995). This, in turn, correlates with the purple ocean hypothesis of Brocks et al., 2005 where C_40_ aromatic carotenoid derivatives such as isorenieratane and okenane detected in mid‐Proterozoic rocks, in addition to their diagenetic breakdown products 2,3,4‐ and 2,3,6‐trimethyl aryl isoprenoids, suggest notable primary productivity by green sulphur bacteria (Chlorobiaceae) and purple sulphur bacteria (Chromatiaceae) where anoxic conditions reached into the photic zone of the water column (e.g., Brocks et al., 2005; Koopmans, Schouten, Kohnen, & Sinninghe Damsté, 1996; Schwark & Frimmel, 2004). Sub‐oxic to anoxic shallow waters and euxinic deep‐water conditions are considered hostile for the expansion of eukaryotes which are largely aerobic and poisoned by sulphide (Anbar & Knoll, 2002). Such conditions were presumably widespread in the mid‐Proterozoic based on biomarker evidence for photic zone euxinia in the 1.64 Ga Barney Creek Formation (Brocks et al., 2005), and the 1.1 Ga Atar Group, Mauritania (Blumenberg, Thiel, Riegel, Kah, & Reitner, 2012; Gueneli, Brocks, & Legendre, 2012), in addition to iron speciation and trace element geochemistry (Canfield, 2004; Poulton et al., 2004) suggested euxinia was a defining feature of the Mesoproterozoic and led to the restriction of eukaryotes to local oases with stable oxygenated conditions.

**Figure 1:**
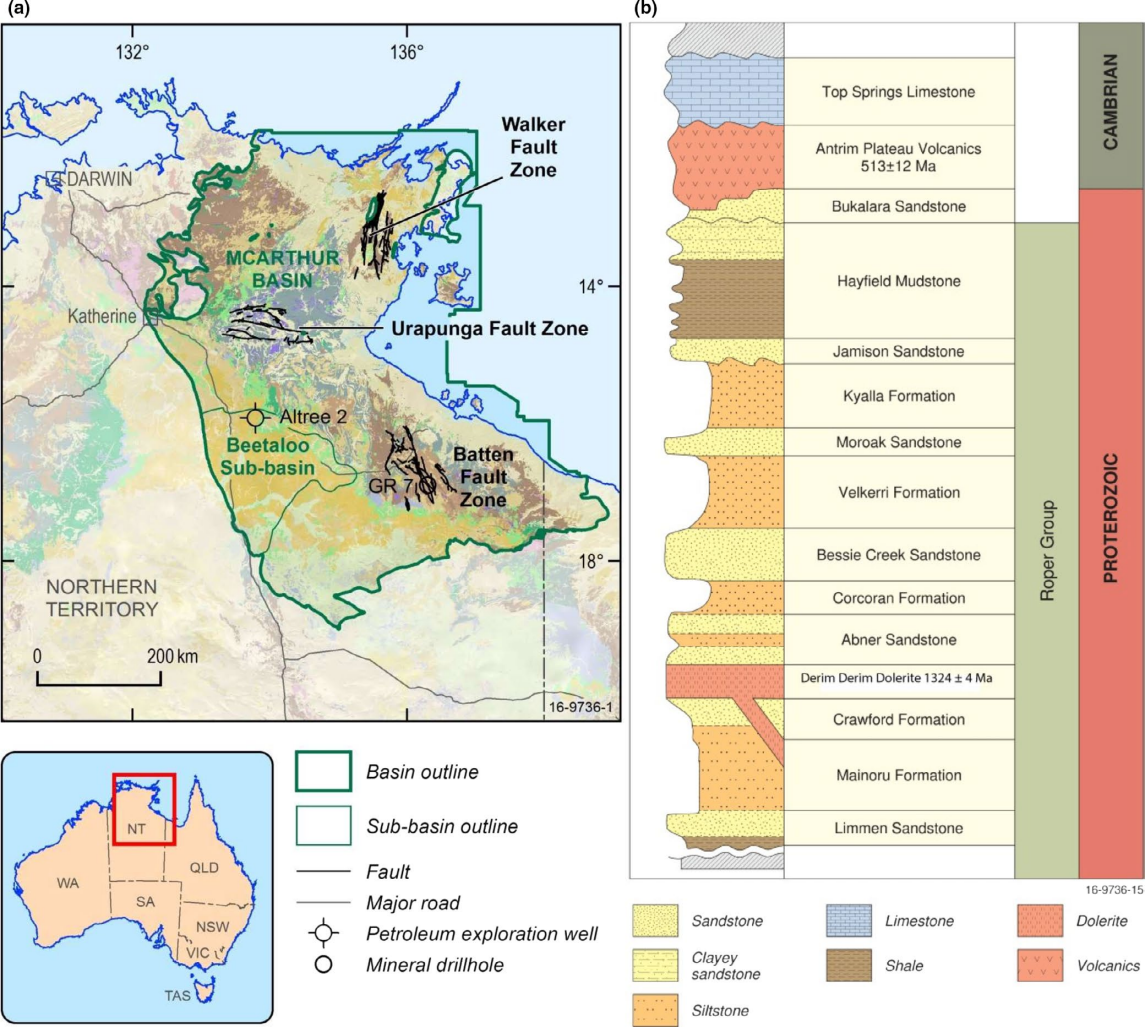
(a) Location map of the McArthur Basin, northern Australia with the Beetaloo Sub basin showing the location of the Altree 2 and the Batten Fault Zone where the GR 7 drillcore is located. (b) Generalised stratigraphy of the Proterozoic Roper Group. Figure adapted from Ahmad et al. (2013). Note that the Derim Derim dolerite sills (not shown) intrude at multiple levels through the Roper Group from the Mainoru Formation through to the base of the Kyalla Formation [Colour figure can be viewed at wileyonlinelibrary.com]

However, in spite of apparent homogeneous and widespread anoxia in deep‐water Mesoproterozoic oceans, redox heterogeneities and fluctuations have been identified from the *ca*. 1.4 Ga Kaltasy Formation, Southern Ural Mountains, Russia, a carbonaceous shale deposited in a basinal, oxic environment (Sperling et al., 2014; Sergeev, Knoll, Vorob’eva, & Sergeeva, 2016); the *ca*. 1.4 Ga Xiamaling Formation of the North China craton, a siliciclastic shale deposited in a low‐energy deep, sub‐tidal environment (Canfield, 2014; Diamond, Planavsky, Wang, & Lyons, 2018), and the age equivalent Velkerri Formation (Cox et al., 2016), Roper Group, northern Australia (Figure 1) suggesting that Mesoproterozoic sequences previously studied may have reflected geochemistry of restricted‐basin settings and contained a taphonomic bias that a larger sample set of biomarker, fossil and geochemistry of different depositional environments including nearshore and oxygenated sedimentary basins may rectify.

**Figure 2:**
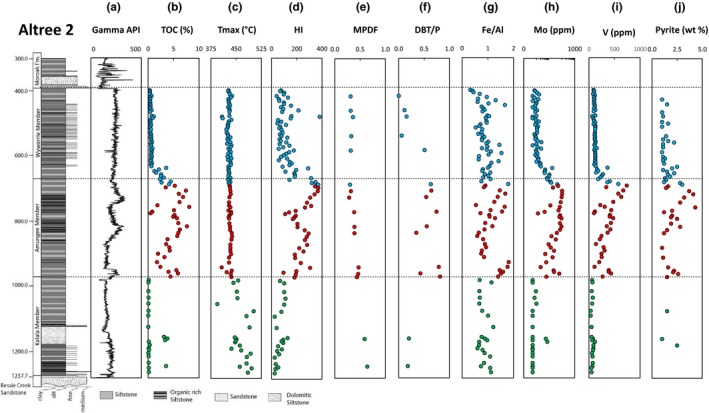
Stratigraphic column of drillcore Altree 2 demonstrating variation in organic and inorganic composition through the Velkerri Formation, A—gamma log (API), B—Total organic carbon (TOC %), C—*T*_max_ (°C), D—Hydrogen Index (HI = (S2/TOC) × 100, mg HC/g rock), E—Methylphenanthrene distribution factor (MPDF = (3 + 2)/(3 + 2+9 + 1)‐methylphenanthrene, integrated in the m/z 192 trace), F—Dibenzothiophene/phenanthrene ratio (DBT/P) integrated in the m/z 184 and 178 traces, G—Iron/aluminium Fe(wt %)/Al(wt %) with a vertical dashed line in the plot at 0.5 to differentiate between oxic and anoxic conditions (Lyons & Severmann, 2006), H—Molybdenum (Mo ppm), shaded area indicates the approximate threshold value for intermittent to persistent euxinia (Lyons et al., 2009), I—Vanadium (V ppm), J—Pyrite (wt %) ppm. Trace element (Al, Fe, Mo, V) and XRD (pyrite wt%) data are replotted from Cox et al. (2016) [Colour figure can be viewed at wileyonlinelibrary.com]

The geochemical structure of the Roper Seaway in the McArthur Basin involved a shallow oxic layer overlying deeper waters that were sub‐oxic to anoxic with intermittent euxinia in the Amungee Member of the Velkerri Formation based on redox‐sensitive trace element data (Figure 2; Cox et al., 2016). Euxinic conditions, as determined by trace element abundances (e.g., enrichment of molybdenum, vanadium and uranium above values typical of the Post Archean Australian Shale), developed once primary productivity was significantly greater than the flux of oxygen, nitrates and oxidised iron, resulting in sulphate reduction (Johnston et al., 2010).

The Roper Group of the McArthur Basin (Figure 1) is also one of the sites that contain the oldest evidence for fossils that have a clear eukaryotic origin (Grey, 2015; Javaux et al., 2001; Peat, Muir, Plumb, McKirdy, & Norvick, 1978). The shoreline facies of the Corcoran and Jalboi formations show the greatest abundance and diversity of biota including the acritarch *Tappania plana* which shows cell organisation and evidence for reproduction via budding that are unique to eukaryotic protists (Javaux, Knoll, & Walter, 2001). Increasing diversity towards the shoreline may be due to either the restriction of bio‐essential trace nutrients to these environs (Anbar & Knoll, 2002) or a taphonomic bias (Javaux et al., 2001). Hydrocarbon biomarkers have a different taphonomic window than fossils (Brocks et al., 2016) and are a complementary proxy to determine whether eukaryotes may have also inhabited distal shelf environments of the Roper Seaway.

Historical studies of biomarkers from the Velkerri Formation focused on its petroleum potential, as the Roper Group contains some of the world's oldest organic‐rich source rocks (TOC content up to 10%) which have generated both oil and gas within the Beetaloo Sub‐basin of the McArthur Basin (Ahmad, Dunster, & Munson, 2013; Jackson, Powell, Summons, & Sweet, 1986; Jackson, Sweet, & Powell, 1988; Summons, Powell, & Boreham, 1988; Figure 1). Saturated hydrocarbons, or biomarkers, extracted from the Velkerri Formation typically included normal alkanes, branched alkanes (including monomethyl alkanes and isoprenoids), triterpanes, hopanes and minor steranes. According to these studies, the high relative concentrations of branched‐alkanes, triterpanes and hopanes imply a bacterial dominated ecosystem, and the low amount of steranes suggests a minor contribution of eukaryotes to the biomass. More recently, it has been acknowledged that anthropogenic hydrocarbon contamination introduced to rock samples during drilling, saw‐cutting and storage can overprint the indigenous hydrocarbon signature (Brocks, Grosjean, & Logan, 2008; French et al., 2015; Grosjean & Logan, 2007). For example, Brocks (2011) measured the spatial distribution of hydrocarbons in 2.7 Ga old Archean shales from the Pilbara in Western Australia. These ancient shales contained indigenous polyaromatic hydrocarbons. A spatial analysis of hydrocarbons between the drill core interior and the outer rounded surfaces revealed saturated hydrocarbons, including steranes and hopanes, were concentrated towards the outer rounded surface of the drill core. Additionally, bulk analyses of dolomitic shales from the 1.64 Ga Barney Creek Formation consistently yielded hopanes and steranes (Summons et al., 1988). However, interior/exterior experiments have concluded that the bulk of the hopanes were indigenous whilst the steranes were contaminants suggesting that the steranes were introduced during drilling and/or storage (Brocks et al., 2008). It has also been shown that fissile shale may be pervasively contaminated within the inner‐core and no amount of cleaning or sawing can remove these contaminants (e.g., Brocks, 2011; Jarrett, Schinteie, Hope, & Brocks, 2013). Thus, the distribution of hydrocarbons between the interior and exterior within samples to detect concentration is crucial prior to biomarker interpretation (Brocks, 2011; French et al., 2015; Jarrett et al., 2013; Schinteie & Brocks, 2014).

A rigorous interior to exterior analysis of indigenous hydrocarbons was recently conducted on samples from the *ca*. 1.38 Ga Velkerri Formation from three drill cores; McManus 1, Shea 1 and Walton 2 with thermal maturity within the oil window (VR equivalent 0.6%–1.3%; Flannery & George, 2014). The study demonstrated that steranes were abundant on the exterior surfaces of the drillcore, though below detection limits in the interior portion, and thus were likely to be contaminants. The small sample size of that study (*n *=* *3) and a relatively high thermal maturity of samples warrants a more detailed investigation into the hydrocarbon composition of the Velkerri Formation, particularly in core going through the oil window. Furthermore, with the recent publication of high‐resolution inorganic geochemistry of the Velkerri Formation in the Altree 2 drillcore (Cox et al., 2016), this study provides a complementary contaminant‐free analysis of hydrocarbon biomarkers preserved in the *ca*. 1.38 Ga Roper Seaway sediments in a palaeoenvironmental context.

## 2 GEOLOGY

### 2.1 Velkerri formation, Roper Group, McArthur Basin

The geology of the Roper Group has been summarised by Abbott and Sweet (2000), Jackson, Muir, and Plumb (1987), and Sweet and Jackson (1986). The Roper Group was deposited in the McArthur Basin, northern Australia, and consists of a *ca*. 1‐ to 5‐km‐thick package of regressive‐transgressive cycles of siliciclastic rocks preserved over *ca*. 145,000 km^2^ with the main depocentre located in the Beetaloo Sub‐basin (Abbott & Sweet, 2000; Plumb & Wellman, 1987; Figure 1). The deeper water Velkerri and Kyalla formations are inferred to be the major source rocks for the oil and gas in the Beetaloo Sub‐basin (Ahmad et al., 2013; Crick, Boreham, Cook, & Powell, 1988; Jackson et al., 1986, 1988; Summons et al., 1988).

Two contrasting tectonic models have been proposed for basin formation and the origin of the Roper Group. One model suggests that the Roper Group was deposited in marine to shelf environments on an epicontinental platform in response to lithostatic extension and sagging (Betts & Giles, 2006; Foster & Ehlers, 1998; Spikings, Foster, Kohn, & Lister, 2001, 2002). A second model suggests that the Roper Group was deposited on an intracratonic ramp that developed during orogenic flexure (Abbott & Sweet, 2000). Both models suggest the Velkerri Formation was deposited in a deep‐water marine environment.

The Velkerri Formation represents a deep‐water, high‐stand systems track. Recently, the Velkerri Formation has been sub‐divided into the Kalala Member (lower), Amungee Member (middle) and upper Wyworrie Members based upon lithology, variations in TOC content, gamma‐ray response and trace metal geochemistry (Munson & Revie, 2018). The Velkerri Formation has been dated by multiple techniques including Rhemium‐osmium (Re‐Os; Kendall, Creaser, Gordon, & Anbar, 2009). Sensitive, high‐resolution ion microprobe (SHRIMP) dates on zircon (Jackson, Sweet, Page, & Bradshaw, 1999) and baddeleyite from the Derim Derim Dolerite (Abbott et al., 2001), and detrital zircon U‐Pb (Yang et al., 2018). Deposition of the Velkerri Formation has been constrained to between 1.38 ± 0.13 Ma (2σ) and 1.36 ± 0.21 Ga (2σ). See Yang et al., 2018 for a detailed discussion on geochronology and provenance of the Roper Group.

The Altree 2 well was drilled in the Beetaloo Sub‐basin in 1998 and chosen for study because it intersects the entire Velkerri Formation from 391.72 to 1,229.65 m (Figure 2). Oil shows were identified through the middle to lower units of the Velkerri Formation and the Bessie Creek Sandstone. An oil stain from 1,126.5 m was analysed by Geoscience Australia (Sample #714) in the Oils of Eastern Australia report (Summons et al., 2002). The report contains “OilMod” parameters for all oils and oil stains in Eastern and Central Australia including ratios of *n*‐alkanes to pristane and phytane, concentrations and ratios of sterane and hopane homologues in addition to δ^13^C_saturates_ and δ^13^C_aromatics_. McArthur Basin oil stains contained low concentrations of steranes and hopanes that were deemed “too low to determine source,” and δ^13^C_saturates_ and δ^13^C_aromatics_ values ranging from ~−32‰ to −34‰, which are isotopically depleted compared to most Phanerozoic oils (Summons et al., 2002).

### 2.2 Samples

In this study, 110 samples from the upper, middle and lower Velkerri Formation of the Altree 2 well were sampled at intervals of approximately 10 m and analysed by Rock‐Eval pyrolysis, X‐ray fluorescence (XRF) and quadrapole inductively coupled plasma mass spectrometry (Q‐ICP‐MS; see Cox et al., 2016). Additionally, 16 thermally immature to mature rocks within the late oil window (*T*_max_ 426–461°C; Figure 2) at approximately 50‐m intervals through the Velkerri Formation (Table 1) were selected for organic extraction and analysis by gas chromatography–mass spectrometry (GC‐MS).

**Table 1:** Samples analysed from the Altree 2 drillcore

GA Sample ID	Member	Depth (mKB)	Sample description	Amount extracted (g)
20150106	Upper Velkerri Fm	410.55	Dark brown to dark grey planar laminated siltstone	11.14
20150115		452.64	Dark grey to black siltstone, flaser‐like cross‐bedding	10.88
20150118		471.24	Dark grey laminated siltstone, silts are surrounded by 2 cm thick sand layers (couplets) with oil shows	10.62
20150129		527.37	Alternating layers of laminated light and dark grey siltstone surrounded by 2 cm thick sand layers (couplets)	8.13
20150138		569.51	Dark grey fissile siltstone surrounded by 20 cm thick sands on either side with oil shows	8.51
20150159		669.51	Dark grey to black laminated siltstone	14.59
20150162	Middle Velkerri Fm	688.61	Dark grey to black pyritic organic‐rich siltstone (white oxidised pyrite on exterior of core)	17.96
20150164		707.51	Black laminated siltstone (white oxidised pyrite on exterior of core)	8.97
20150169		750.28	Black laminated siltstone	20.33
20150175		793.37	Black laminated pyritic siltstone	13.42
20150177		812.11	Black to dark grey laminated siltstone	8.16
20150186		912.37	Dark grey to dark brown massive siltstone	11.78
20150189		931.33	Black laminated pyritic siltstone (white oxidised pyrite on exterior of core)	13.88
20150190		940.69	Black laminated siltstone	4.65
20150197	Lower Velkerri Fm	1,122.40	Black to dark brown laminated siltstone	14.77
20150209		1,203.28	Greyish black highly fissile siltstone	8.41

## 3 METHODS

The methods for inorganic and organic geochemistry have been described in detail elsewhere (Boreham & Ambrose, 2007; Cox et al., 2016; Jarrett et al., 2013; Lee & Brocks, 2011). TOC measurements and Rock‐Eval pyrolysis measurements were determined on powdered rock samples via pyrolysis using a Rock‐Eval 6^™^ instrument. Rocks were treated with a rigorous screening technique to determine and remove surficial contamination (e.g., Jarrett et al., 2013). Powdered rock samples were extracted using organic solvents and analysed by GC‐MS using a Hewlett Packard HP 6890 gas chromatograph interfaced to an HP 5975 mass spectrometer in full scan mode (Boreham & Ambrose, 2007). Metastable Reaction Monitoring (MRM) analyses were performed using an Agilent 6890 gas chromatograph interfaced to a Micromass Autospec Premier double sector mass spectrometer (Jarrett et al., 2013). All samples analysed by MRM were injected in *n*‐hexane to avoid FeCl_2_ build‐up in the MS ion source and subsequent deterioration of chromatographic signals through the use of halogenated solvents (Brocks & Hope, 2014).

Ancient rocks can be affected by trace amounts of contamination potentially altering the indigenous hydrocarbon signature (Brocks et al., 2008; Jarrett et al., 2013). Laboratory system blanks covering the sawing, crushing, extraction, fractionation and analysis process contained near‐zero hydrocarbon contamination background levels. Using rigorous assessments for syngeneity as described previously (e.g., Brocks et al., 2008), the 16 interior fractions contain indigenous biomarkers. Only, these untainted extracts are investigated further.

The δ^13^C of individual *n*‐alkanes were measured by gas chromatography–combustion–isotope ratio mass spectrometry (GC‐C‐IRMS) using a Thermo Scientific MAT 253 isotope ratio mass spectrometer. Briefly, the hydrocarbon gas components were chromatographically separated using a DB‐5 capillary column (60 m × 0.32 mm ID. Film thickness 0.25 μm; Agilent) with ultra‐high‐purity (UHP) helium as a carrier gas at a constant flow rate of 2.0 ml/min. The on‐column injector temperature was held at 150°C. The 2 μl of pentane solution with sample *n*‐alkanes was injected either manually or with the GC PAL autosampler. The δ^13^C values are reported relative to VPDB. All analyses were run as a minimum in duplicate with a maximum experimental error of 0.5‰.

## 4 RESULTS AND DISCUSSION

### 4.1 Bulk characteristics

The bulk characteristics of the Velkerri Formation in Altree 2 are published by Cox et al. (2016) for all samples plotted in Figure 2. Within the Altree 2 drillcore, the TOC content ranges from 0.04% to 8.1% (*n *=* *112, avg. = 1.96%, *SD* = 2.17). These results replicate those previously reported for the Velkerri Formation in Altree 2 (Jackson et al., 1988) and McManus 1 (Warren, George, Hamilton, & Tingate, 1998), with the middle Velkerri Formation (Amungee Member) containing the highest average TOC content (*n *=* *31, avg. = 4.63%, *SD* = 0.7%; Figure 2). The organic richness and quality, as measured by the S1, S2 and hydrogen index (HI; Figure 2) parameters, show three maxima, all of which are hosted within the Amungee Member.

*T*_max_ values increase downcore from 427°C at 394.1 m to 485°C at 1,220.65 m (Figure 2). The onset of oil generation in the McArthur Basin has been calculated to occur at ~435°C and the end of the oil window at 470°C (Crick, 1992; Crick et al., 1988). Therefore, *T*_max_ values for the upper Velkerri Formation suggest thermal immaturity (*n *=* *58, avg. = 428°, *SD* = 5.3), with the middle Velkerri Formation exhibiting maturity levels that range from immature, to the onset of hydrocarbon generation (435°C at 765 m: Figure 2) into the main oil window (440°C at 950 m; Figure 2) close to the base of the middle Velkerri Formation (*n *=* *31, avg. = 431°, *SD* = 5.6). The lower Velkerri Formation is thermally mature within the late oil window (*n *=* *22, avg. = 463°, *SD* = 25.3°) co‐incident with the oil staining identified through the lower sections of the core. The intrusion of a dolerite sill at 1,688 m, into the Bessie Creek Sandstone unit at the base of Altree 2 (Crick, 1992; Warren et al., 1998), has locally increased the thermal maturity.

The hydrogen index (HI mg/g TOC) in Altree 2 displays different trends for the three members of the Velkerri Formation. The Wyworrie Member in the upper Velkerri Formation has HI values that increase downcore from 75 to 140 mg/g TOC (HI average 130 mg/g TOC; stdev 83). The Amungee Member has three fluctuations in HI broadly consistent with gamma API and TOC (HI average 239 mg/g TOC; stdev 72), and the underlying Kalala Member has HI values between 100 and 20 mg/g TOC (HI average 67 mg/g TOC; stdev 31). An increase in TOC at a dolomitic siltstone layer between 1,117 and 1,127 m correlates with an increase in HI. Variations in HI in Altree 2 appear to be influenced by both initial TOC and thermal maturity (Figure 2). In the Wyworrie Member and the upper Amungee Member, HI and TOC correlate. After the onset of hydrocarbon generation in the lower Amungee Member, the HI broadly decreases downcore due to expulsion of hydrocarbons (Bordenave, 1993).

### 4.2 Saturated hydrocarbons

**Figure 3:**
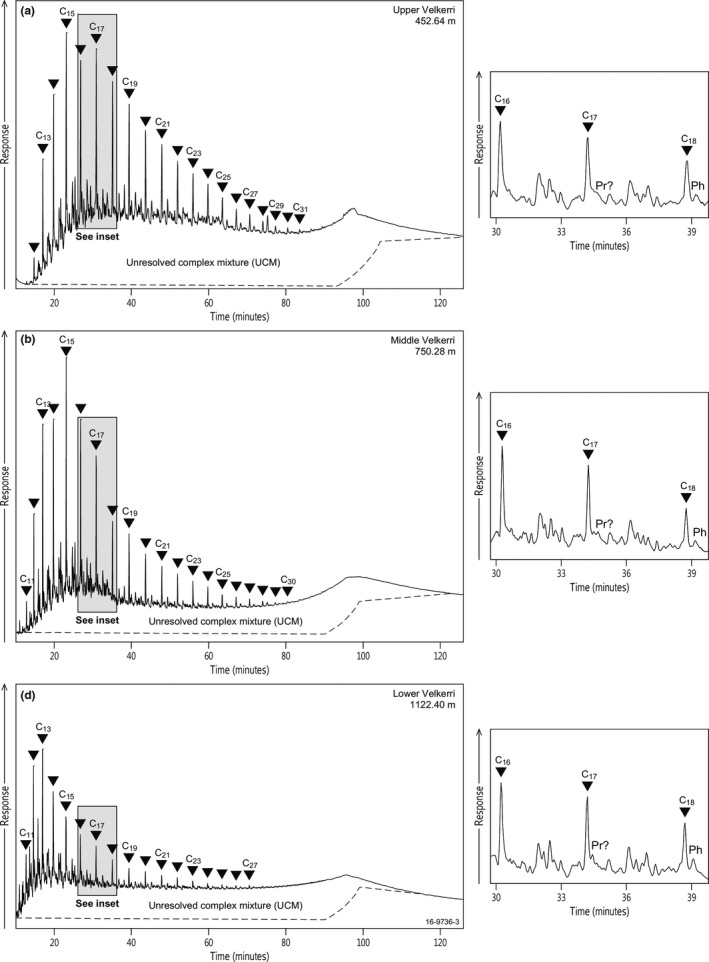
GC‐MS full scan total ion chromatograms (TIC) of the saturated hydrocarbon fractions of representative samples from (a) the upper Velkerri Formation, 452.64 m, (b) middle Velkerri Formation, 750.28 m, and (c) lower Velkerri Formation, 1,122.40 m in the Altree 2 drillcore. Triangles represent n‐alkanes, Pr: pristane; Ph: phytane; UCM: unresolved complex mixture; MMAs: Monomethyl alkanes

Total ion currents of the saturated hydrocarbon fraction for representative samples from the Wyworrie, Amungee and Kalala members of the Velkerri Formation are illustrated in Figure 3. All samples analysed are characterised by a large unresolved complex mixture (UCM) and a higher than usual proportion of monomethyl alkanes relative to regular *n*‐alkanes (Figure 3), such as is typical of indigenous Proterozoic hydrocarbons (Pawlowska, Butterfield, & Brocks, 2013). All extracts of the Velkerri Formation contain *n*‐alkanes from C_11_ to at least C_27_, with a maximum at either C_13_ or C_15_, and an odd‐over‐even predominance of carbon numbers in the range C_12_–C_18_. This odd‐carbon numbered predominance is also a typical feature in many Ordovician bitumens and oils, where a plausible source is the phototrophic micro‐organism *Gloeocapsomorpha prisca* (Fowler, 1992 and references therein; Peters, Walters, & Moldowan, 2005). However, other studies have shown that this feature is generally typical in cyanobacteria (Evans, 1994; Lea‐Smith et al., 2015; Peters et al., 2005), which is the most likely source of the *n*‐C_13_, *n*‐C_15_ and *n*‐C_17_ predominance in the Velkerri Formation.

Monomethyl alkanes were detected in all samples including both mid‐chain and 2‐methyl and 3‐ methyl (iso‐ and anteiso‐) terminal branching isomers with similar carbon number distribution as the *n*‐alkanes. This is similar to the work by Summons et al. (1988) who used MRM to characterise the monomethyl alkanes in the Velkerri and Barney Creek formations. Summons et al. (1988) identified that the Velkerri Formation contained mid‐chained MMAs dominated in low molecular weight ranges (C_13_–C_19_), and the terminal branching isomers became predominant with higher molecular weights. Immature Barney Creek Formation extracts contain high ratios of terminal branching isomers compared to mid‐chained isomers, and these reduce to comparable abundances in the more mature samples (Summons et al., 1988). Regularly branched isoprenoids up to C_20_, including pristane (Pr) and phytane (Ph), are tentatively identified in trace concentrations in all samples (Figure 3), while irregular isoprenoids such as crocetane and regular C_20+_ acyclic isoprenoids are below detection limits based on relative elution positions with standards. These results are similar to Summons et al. (1988) who identify the major difference between the Velkerri Formation and underlying Barney Creek Formation is the absence of C_20+_ isoprenoids. Low concentrations of isoprenoids are characteristic of the biomarker “Proterozoic facies 1” of Pawlowska et al. (2013) as exemplified by the 1.1 Ga Neryuen Formation of the Sette‐Daban fold belt, Siberia, the 1.1 Ga Nonesuch Formation of the Mid Continent Rift System, North America and deep sections of the 1.64 Ga Barney Creek Formation of the McArthur Group (Pawlowska et al., 2013; Pratt, Summons, & Hieshima, 1991; Summons et al., 1988). Saturated carotenoids, including β‐carotene, and associated carotane cleavage products were not detected in the *m/z* 125–123 chromatogram in any of the extracts in this study based on comparison of elution positions with the Barey Creek Formation (Lee & Brocks, 2011).

### 4.3 Steranes and hopanes

**Figure 4:**
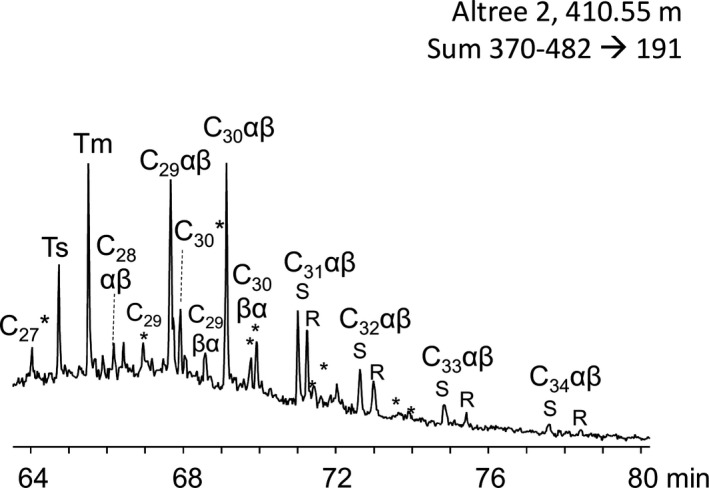
Distribution of the C27 to C34 regular hopanes in the combined m/z 370, 384, 398, 412, 426, 440, 454, 468, 482 (370–482) → 191 traces in the upper Velkerri Formation—drillcore Altree 2, 410.55 m. Peak assignments define the C‐22 stereochemistry (R and S), ɑβ indicates 17ɑ(H),21β(H), Ts = C27 18ɑ(H),22,29,30‐trisnorneohopane, Tm = C27 17ɑ(H),22,29,30‐trisnorhopane and * = diahopanes

Regular steranes, diasteranes and gammacerane, biomarkers commonly used as proxies for eukaryotes (Peters et al., 2005), were below detection limits in all samples even using sensitive MRM analysis (Figure 4). By contrast, C_27_ to C_35_ regular hopanes and their diagenetically rearranged isomers (diahopanes) were detected in the Wyworrie and Amungee Members from 410.55 to 793.37 m with the dominant hopanes being the regular C_30_ αβ and C_29_ αβ hopanes (Figure 4). The concentration of regular hopanes declined downcore from 3.8 ng/g rock at 410.55 m to 1.1 ng/g rock at 793.37 m (Table 2). Below this depth, hopanes were beneath detection limits. At that depth, the thermal maturity increases to the peak oil window (*T*_max_ 432°C; Rc(MPDF) 0.70%; Table 2), suggesting that the decreasing hopane abundances downcore in Altree 2 reflect thermal breakdown. The homohopane index (C_35_/∑C_31_–C_35_ hopanes) is a measure of hopane sulphurisation. Under oxic conditions, it has been shown that the C_35_ hopanepolyol side chain can degrade, generating lower molecular weight hopanes, while sulphurisation will preferentially preserve C_35_ homologue (Peters et al., 2005). The homohopane index is <0.15 for all samples analysed which may indicate the absence of sulphurisation in the Wyworrie and Amungee Members (Table 2). However, with the onset of thermal maturity, C_31_ is preferentially desulphurised, limiting the proxy to immature sediments (Köster, Van Kaam‐Peters, Koopmans, De Leeuw, & Sinninghe Damsté, 1997; Peters et al., 2005). Thus, limiting the utility of the homohopane index in the Amungee Member.

**Table 2:** Rock‐Eval pyrolysis and biomarker parameters for indigenous interior extracts in drillcore Altree 2

Member	Upper Velkerri Fm						Middle Velkerri Fm								Lower Velkerri Fm	
Depth (m)	410.55	452.64	471.24	527.37	569.51	669.51	688.61	707.51	750.28	793.37	812.11	912.37	931.33	940.69	1,122.40	1,203.28
TOC (%)^a^	0.68	0.68	1.13	0.61	0.58	3.07	4.13	7.45	6.85	7.63	0.68	0.49	0.46	0.56	0.62	0.55
*T*_max_ (°C)^b^	438	431	408	426	431	430	433	430	435	432	437	407	434	436	451	461
Sum of hopanes^c^	3.77	3.84	1.99	2.66	2.02	1.42	3.45	8.78	6.14	1.14	n.d.	n.d.	n.d.	n.d.	n.d.	n.d.
Sum of steranes^d^	n.d.	n.d.	n.d.	n.d.	n.d.	n.d.	n.d.	n.d.	n.d.	n.d.	n.d.	n.d.	n.d.	n.d.	n.d.	n.d.
Ts/(Ts + Tm)^e^	0.35	0.57	0.49	0.60	0.62	0.62	0.65	0.61	0.62	0.49	n.d.	n.d.	n.d.	n.d.	n.d.	n.d.
C_29_ ɑβ/(ɑβ + βɑ)^f^	0.78	0.86	0.91	0.52	0.65	1.00	0.36	0.76	0.61	1.00	n.d.	n.d.	n.d.	n.d.	n.d.	n.d.
C_31_ S/(S + R)^g^	0.51	0.52	0.51	0.50	0.51	0.38	0.54	0.49	0.57	0.25	n.d.	n.d.	n.d.	n.d.	n.d.	n.d.
C_31_/C_30_ h	0.71	0.82	0.64	0.78	0.72	0.90	0.55	1.43	1.52	0.17	n.d.	n.d.	n.d.	n.d.	n.d.	n.d.
C_35_/(C_30_–C_35_)	0.05	0.11	0.06	n.a.	n.a.	n.a.	0.08	n.a.	0.08	n.a.	n.d.	n.d.	n.d.	n.d.	n.d.	n.d.
C_31_ 2α MeHop	0.05	n.d.	0.02	n.d.	n.d.	n.d.	n.d.	n.d.	n.d.	n.d.	n.d.	n.d.	n.d.	n.d.	n.d.	n.d.
C_31_ 3β MeHop	0.03	n.d.	0.01	n.d.	n.d.	n.d.	n.d.	n.d.	n.d.	n.d.	n.d.	n.d.	n.d.	n.d.	n.d.	n.d.
MPDF[Link]	0.31	0.31	0.36	0.33	0.32	0.30	0.32	0.28	0.38	0.39	0.39	0.47	0.46	0.44	0.59	0.64
Rc(MPDF)^j^	0.54	0.54	0.64	0.56	0.54	0.51	0.55	0.47	0.69	0.70	0.70	0.89	0.87	0.82	1.16	1.27
MPR^k^	0.57	0.70	0.79	0.64	0.78	0.61	0.70	0.64	0.94	1.03	1.09	1.30	1.17	0.88	2.24	2.26
Rc(MPR)^l^	0.69	0.79	0.84	0.75	0.83	0.73	0.79	0.75	0.91	0.95	0.98	1.05	1.01	0.89	1.29	1.29
DBT/P^m^	0.00	0.11	0.15	0.06	0.51	0.63	0.64	0.54	0.75	0.55	0.35	0.79	0.43	0.81	0.20	0.18

Notes

n.a.: not analysed, compete set of C_31_–C_35_ homologues below detection limits; n.d.: not detected.

^a^Total Organic Carbon (%). ^b^ *T*_max_ (°C). ^c^Sum of the C_27_ to C_35_ hopanes (Ts + Tm + C_28_–C_35_ αβ 22R + 22S isomers, ng/g extracted rock) (Moldowan, Seifert, & Gallegos, 1985). ^d^Sum of the steranes (ng/g rock) calculated using the ααα and αββ 20S + 20R isomers for C_27_–C_30_ steranes (Seifert & Moldowan, 1986). ^e^Ts/(Ts + Tm) (McKirdy, Alridge, & Ypma, 1983). ^f^C_29_ hopane ratio (C_29_ αβ/(αβ + βα) (Seifert & Moldowan, 1986). ^g^C_30_ hopane ratio (C_30_ αβ/αβ + βα) (Seifert & Moldowan, 1986). ^h^Hopane ratio = C_31_ αβ (22R + 22S)/C_30_ (αβ) (Seifert & Moldowan, 1980). ^i^Methylphenanthrene distribution factor MPDF = (3‐MP + 2‐MP)/(3‐MP + 2‐MP + 9‐MP + 1‐MP) (Boreham et al., 1988). ^j^Calculated vitrinite reflectance equivalent based on the MPDF (Boreham et al., 1988) Rc(MPDF) = 2.242(MPDF)−0.166. ^k^Methylphenanthrene ratio MPR = 2‐MP/1‐MP (Radke et al., 1990). ^l^Calculated vitrinite reflectance based on the MPR (Rc(MPR) = 0.99 log_10_MPR + 0.94) (Radke et al., 1990). ^m^Dibenzothiophene/phenanthrene integrated in *m/z* 184 and 178 TIC (Hughes et al., 1995).

**Figure 5:**
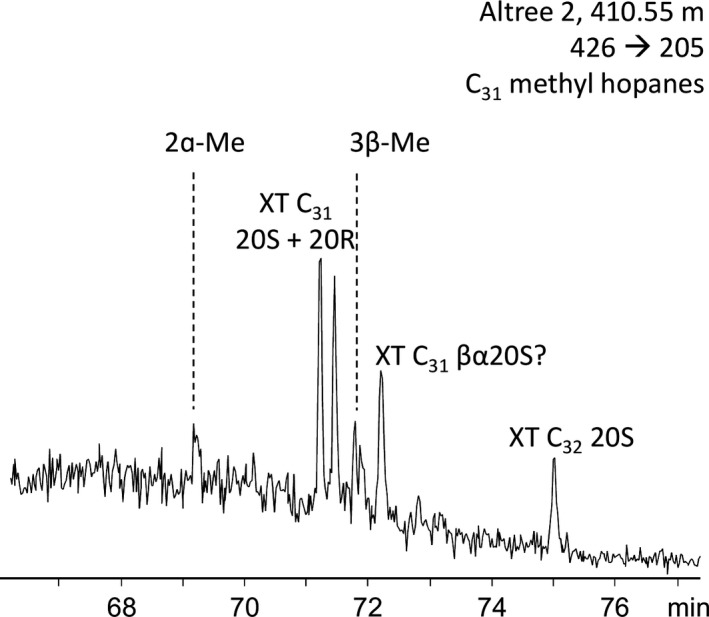
Representative MRM m/z 426 > 205 mass fragmentogram in the upper Velkerri Formation—drillcore Altree 2, 410.55 m showing relative abundances of A‐ring methylated hopanes

C_31_ 2α‐ and 3β‐methylhopanes were detected in trace concentrations in two samples in the Wyworrie Member and were below detection limits in the Amungee and Kalala Members (Figure 5; Table 2). The C_31_ 2α‐methylhopane index (MHI) ranges from 8.5% to 6.6% (Table 2) and the 3β‐methylhopane index from 5.1% to 4.5% (Table 2). Trace concentrations of 2α‐ and 3β‐methylhopanes were also identified in Velkerri Formation samples analysed by Flannery and George (2014). The presence of 2α‐methylhopanes (2α‐MHI 3% to 7%) may be complimentary evidence for the presence of cyanobacteria (Summons, Jahnke, Hope, & Logan, 1999), although these molecules are also produced by a range of other bacteria (Ricci et al., 2013; Welander, Coleman, Sessions, Summons, & Newman, 2010). The presence of 3β‐methylhopanes may indicate the presence of methanotrophic bacteria (Farrimond, Talbot, Watson, Schulz, & Wilhelms, 2004; Peters et al., 2005). Methanotrophic bacteria often inhabit the boundary between anoxic and oxic conditions and are particularly active in water bodies with low sulphate content (Beal, Claire, & House, 2011; Farrimond et al., 2004; Hanson & Hanson, 1996). Inorganic geochemistry is consistent with this preferred environment for methanotrophs. The water column during deposition of the Wyworrie Member is predominantly sub‐oxic to anoxic at depth (Cox et al., 2016).

### 4.4 Compound‐specific isotopic analysis (CSIA) of *n*‐alkanes

**Figure 6:**
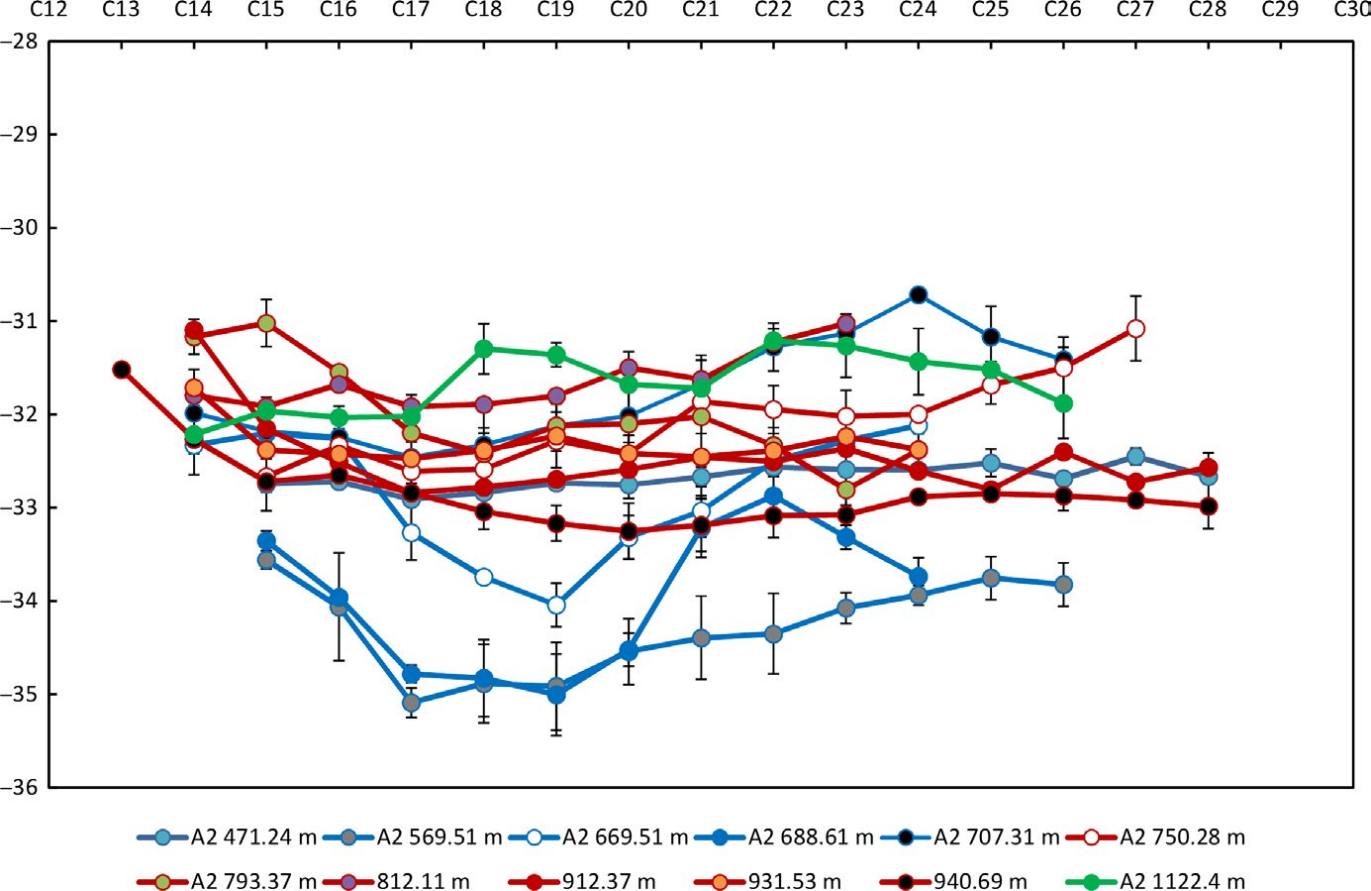
Distribution of δ13C values of n‐C13 to n‐C28 for upper (blue lines and diamonds), middle (green lines and circles) and lower (red line and triangles) Velkerri Formation samples from Altree 2 [Colour figure can be viewed at wileyonlinelibrary.com]

Carbon compound‐specific isotopic analyses (CSIA; δ^13^C) of individual *n*‐alkanes are presented in Figure 6 and Table 3. Values range from −31‰ to −35‰ with an average isotopic enrichment downcore (Figure 6). The average value for the Wyworrie Member is −33.3‰, the Amungee Member is −32.2‰, and the Kalala Member is −31.6‰ (Table 3). Isotopic enrichments can be caused by thermal maturity (Clayton, 1991; Tang et al., 2005) leading to downcore trends in δ^13^C enrichment. The presence of the Derim Derim sill in the lower Altree 2 (1,688 m) would provide additional heat flow in the middle to lower Velkerri (Clayton & Bostick, 1986; Meyers & Simoneit, 1999; Simonet, Brenner, Peters, & Kaplan, 1981). However, there is no steady downcore increase in the δ^13^C enrichment with depth suggesting that organic source, or level of recycling of the organic matter, is a greater influence on carbon isotopes than thermal maturity. Many samples show a trend of enrichment of the shorter (<*n*‐C_20_) chained *n*‐alkanes (Figure 6), a trend that has been shown to occur due to heterotrophic reworking of organic matter (Hayes, 2001; Schouten et al., 1998). Alternatively, oil staining has been identified in Altree 2 at 669.51 m with a high S1 peak and low *T*_max_ value (Cox et al., 2016) suggesting a migration of low molecular weight hydrocarbons into the system. However, evidence for oil migration is not evident at 569 and 688 m where strong short‐chain *n*‐alkane enrichment is particularly evident.

**Table 3:** *n*‐Alkane carbon isotopic composition (δ^13^C in ‰ vs PDB) for Velkerri Formation samples from Altree 2

GA Sample ID	Member	Depth (m)	Carbon number															
			13	14	15	16	17	18	19	20	21	22	23	24	25	26	27	28
2015011801	Upper Velkerri	471.24			−32.75	−32.72	−32.91	−32.84	−32.73	−32.76	−32.67	−32.57	−32.59	−32.59	−32.52	−32.69	−32.45	−32.67
2015013801		569.51			−33.56	−34.06	−35.09	−34.89	−34.91	−34.54	−34.39	−34.35	−34.08	−33.94	−33.75	−33.82		
2015015901		669.51		−32.33	−32.20	−32.26	−33.27	−33.74	−34.04	−33.32	−33.03	−32.50	−32.28	−32.12				
2015016201		688.61			−33.35	−33.96	−34.78	−34.83	−35.00	−34.52	−33.22	−32.87	−33.32	−33.74				
2015016401	Middle Velkerri	707.51		−31.99	−32.18	−32.24	−32.46	−32.33	−32.12	−32.02	−31.67	−31.28	−31.13	−30.72	−31.17	−31.41		
2015016901		750.28			−32.67	−32.33	−32.61	−32.59	−32.28	−32.42	−31.86	−31.95	−32.02	−32.00	−31.68	−31.50	−31.08	
2015017501		793.37		−31.17	−31.02	−31.55	−32.20	−32.42	−32.12	−32.10	−32.02	−32.33	−32.80	−32.38				
2015017701		812.11		−31.80	−31.92	−31.68	−31.91	−31.89	−31.80	−31.50	−31.62	−31.22	−31.02					
2015018601		912.37		−31.09	−32.15	−32.51	−32.84	−32.78	−32.69	−32.59	−32.46	−32.50	−32.36	−32.61	−32.81	−32.40	−32.72	−32.57
2015018901		931.33		−31.71	−32.38	−32.43	−32.47	−32.39	−32.23	−32.42	−32.45	−32.39	−32.24	−32.38				
2015019001		940.69	−31.52	−32.27	−32.72	−32.65	−32.84	−33.04	−33.17	−33.25	−33.19	−33.09	−33.08	−32.88	−32.85	−32.87	−32.92	−32.98
2015019701	Lower Velkerri	1,122.40		−32.22	−31.96	−32.03	−32.02	−31.30	−31.36	−31.68	−31.72	−31.21	−31.26	−31.43	−31.52	−31.88		

### 4.5 Aromatic hydrocarbons

Thermal maturity parameters based on the methylphenanthrene distribution factor (MPDF) and methylphenanthrene ratio (MPR; Boreham, Crick, & Powell, 1988; Radke, Garrigues, & Willsch, 1990; Radke, Willsch, & Leythaeuser, 1982) increase with depth ranging from Rc (MPDF) = 0.54%–1.27% (Table 2). These values correspond to the onset of the oil window (Rc ~0.6%) to the late oil window (Rc ~1.8%) and are similar to thermal maturities based on aromatic hydrocarbons in Altree 2 (Summons, Taylor, & Boreham, 1994) and in other wells through the Velkerri Formation (Boreham et al., 1988; Warren et al., 1998; George & Ahmed, 2002). George & Ahmed (2002) undertook a comprehensive study of 16 different aromatic maturity parameters in three wells in the Velkerri Formation. The methylphenanthrenes were shown to be the most sensitive to maturity variations from thermally immature to late oil window (George & Ahmed, 2002). This provides confidence in the thermal maturity results in this study. Additionally, the aromatic thermal maturity values correlate with *T*_max_ values ranging from 438°C, onset of oil generation, to 461°C towards the end of the oil window (Table 2; Figure 2). The dibenzothiophene to phenanthrene (DBT/P) ratio is <1 for all Velkerri Formation samples analysed in this study (Figure 2), consistent with values typical for shales (Hughes, Holba, & Dzou, 1995). Additionally, there are two clusters of DBT/P ratios in the extracts; the first cluster, comprising Kalala and Wyworrie Member, contains low relative abundances of S and low DBT/P ratios (Figure 2). The Amungee Member has higher S contents and elevated DBT/P ratios probably due to the incorporation of reduced sulphur (H_2_S) into the organic matter. Increases in DBT have been shown to broadly correlate with increasing sulphide concentrations in the deep‐water column that is then in turn incorporated into the organic matter (Hughes et al., 1995; Werne et al., 2008). Therefore, these aromatic hydrocarbons may provide evidence for deep‐water euxinia in the middle Velkerri Formation. Such high DBT/P ratios occur at the same depths as previously published inorganic proxies suggesting deep‐water euxinia in the middle Velkerri Formation (Cox et al., 2016 and Figure 2).

**Figure 7:**
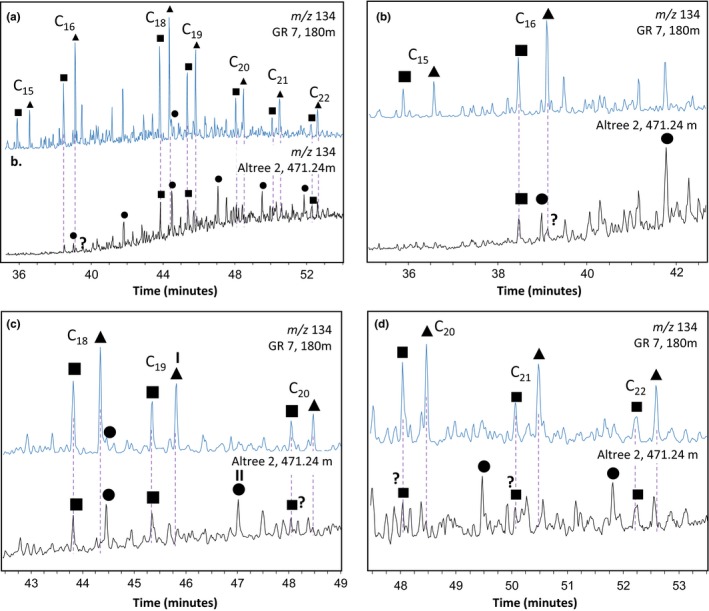
(a) Partial m/z 134 mass chromatogram comparing the Barney Creek Formation from drill core GR 7 at 180 m (blue) to the Velkerri Formation in Altree 2 at 471.24 m (black). Magnification of time series demonstrate the relative elution positions of the trimethyl aryl isoprenoids and the trimethyl alkylbenzenes between (b) C15–C17, (c) C18–C20 and (d) C20–C22. Squares, 2,3,6‐trimethyl aryl isoprenoids; triangles, 2,3,4‐trimethyl aryl isoprenoids; circles, trimethyl alkylbenzenes. I is C19 2,3,4 trimethyl aryl isoprenoid and II is C19 trimethyl alkylbenzene. Note the two chromatograms are not 1:1 as Altree 2 has been significantly magnified [Colour figure can be viewed at wileyonlinelibrary.com]

**Figure 8:**
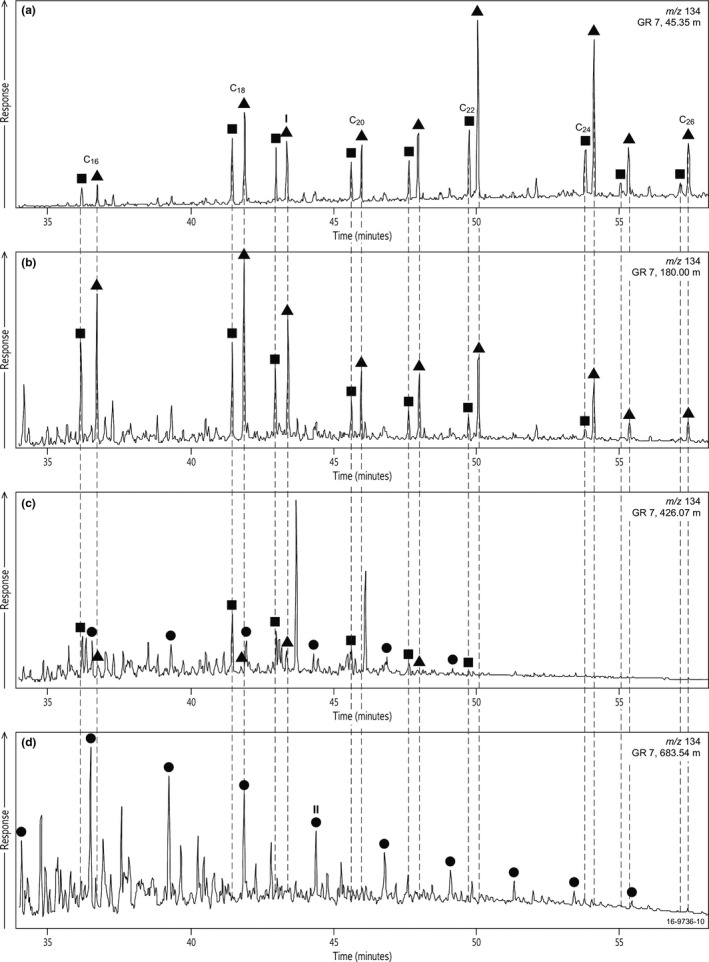
Partial m/z 134 mass chromatogram comparing samples from the Barney Creek Formation in drillcore GR 7 with different depths. Squares, 2,3,6‐trimethyl aryl isoprenoids; triangles, 2,3,4‐trimethyl aryl isoprenoids; circles, trimethyl alkylbenzenes. I is C19 2,3,4‐trimethyl aryl isoprenoid and II is C19 trimethyl alkylbenzene

Intact C_40_ aromatic carotenoids such as isorenieratane and okenane are commonly used as biomarkers for green sulphur bacteria (Chlorobiaceae) and purple sulphur bacteria (Chromatiaceae), which thrive under conditions of photic zone euxinia (PZE) (Schaefle, Adam, Wehrung, & Albrecht, 1997; Grice, Schaeffer, Schwark, & Maxwell, 1996; Koopmans et al., 1996; Summons & Powell, 1987; Brocks et al., 2005). Intact C_40_ carotenoids were not detected in any of the Altree 2 extracts. However, aromatic carotenoids are commonly cleaved into shorter fragments during diagenesis, yielding 2,3,4‐trimethyl aryl isoprenoids (TMAI) as the breakdown products of okenone and other carotenoids of purple sulphur bacteria, and 2,3,6‐TMAI as the cleavage products of chlorobactene and isorenieratene found in green sulphur bacteria (Brocks & Summons, 2003; Koopmans et al., 1996; Schwark & Frimmel, 2004). Figure 7 investigates the presence or absence of 2,3,4‐ and 2,3,6‐TMAI in the Velkerri Formation by juxtaposing *m/z* 134 partial mass chromatograms of an Altree 2 samples (black trace) with bitumen from the older Barney Creek Formation where these biomarkers are abundant (blue trace; Brocks et al., 2005). In such comparisons, it is important to recognise that successful identification of TMAI relies on the presence of a continuous pseudohomologous series with plausible relative abundances, and that many of the short‐ to medium‐chain length pseudohomologues (e.g., C_11_–C_18_) may coelute with trimethylbenzenes (TMAB) and other interfering compounds (Figure 8). For the Altree 2 sample in Figure 7, a complete series of 2,3,6‐TMAI appears to be present in the range C_16_–C_22_. However, the concentration of these compounds is low and their identification must remain tentative due to the presence of numerous interfering signals. The Altree 2 sample also has a few peaks at the right elution position for the 2,3,4‐TMAI series. However, there is virtually no signal for the C_18_ and C_21_ pseudohomologs, so the entire series must be interpreted as not detected.

The genuine absence or low concentration of TMAI in the Velkerri Formation is important because it distinguishes its environment from other Proterozoic formations such as the Barney Creek Formation (Brocks et al., 2005) and the 1.1 Ga El Mreïti Group (Gueneli et al., 2018). We therefore investigate whether the extremely low concentrations of TMAI reflect primary absence or are due to thermal breakdown or oxygen exposure during degradation.

To determine whether 2,3,4‐ and 2,3,6‐TMAI were removed from Velkerri Formation bitumens by thermal breakdown, maturity proxies from the Altree 2 drillcore were compared with a Barney Creek Formation profile from drillcore GR 7 (Figure 8). The Barney Creek Formation in the GR 7 drillcore is composed of dolomitic mudstones and siliciclastic shales that range in maturity from immature (*T*_max_ 430°C at 50.63 m) to the peak oil zone at the base of the drillcore (*T*_max_ 446°C at 854.30 m; Lee & Brocks, 2011; Revie, 2016). Thermal breakdown downcore of β‐carotene, C_40_ aromatic carotenoids and 2,3,4‐ and 2,3,6‐TMAIs have been previously reported in GR 7 (Lee & Brocks, 2011). These authors documented that intact β‐carotane was preserved to *ca*. 140 m, while β‐carotane breakdown fragments were identified downcore to *ca*. 290 m. Additionally, in the current study of GR 7, 2,3,4‐ and 2,3,6‐TMAIs are abundant at shallow depths (e.g., 45.35 m; Figure 8), while TMAI decrease and TMABs become relatively more abundant with increasing depth and thermal maturity (Figure 8). The C_15_ 2,3,6‐TMAI/TMAB ratio decreases from 6.7 at 45.35 m to 0.11 at 869.60 m, and C_18_ 2,3,6‐TMAI/TMAB decreases from 15.1 to 0.36 over the same depth range. The C_15_ and C_18_ 2,3,6‐TMAI/TMAB ratios fall below 1 by 426.7 m (*T*_max_ 434°C) suggesting that in GR7 the ratio reaches unity at the onset of the oil window. The detection of TMAIs is sensitive to thermal maturity, and the presence of phototrophic sulphur bacteria and PZE conditions can presumably only be detected reliably at immature to moderate thermal maturities, at least in Proterozoic organic facies.

In Altree 2, thermally immature *T*_max_ values < 434°C are encountered to a depth of nearly 1,000 m (Figure 2). Yet, intact C_40_ carotenoids and the associated cleavage products are below detection limits. Tentative identified 2,3,6‐TMAIs (C_16_,C_19_,C_20_,C_22_) only occur in traces (Figure 7). Apart from the Barney Creek Formation, numerous studies have identified intact C_40_ carotenoids and TMAIs in immature to mature samples in the early oil window (e.g., Grice et al., 1996; Koopmans et al., 1996; Schwark & Frimmel, 2004). Therefore, the absence of clearly identified TMAI in the Amungee and Wyworrie Members cannot be a pure thermal breakdown effect but is presumably an ecological or diagenetic phenomenon. Consequently, either the Roper Basin was never inhabited by phototrophic sulphur bacteria because anoxic waters never reached into the photic zone, or the biomarkers of these organisms were destroyed by heterotrophic reworking, most likely through (intermittent) exposure to oxygen at the sediment‐water interface (e.g., Repeta, 1989).

**Figure 9:**
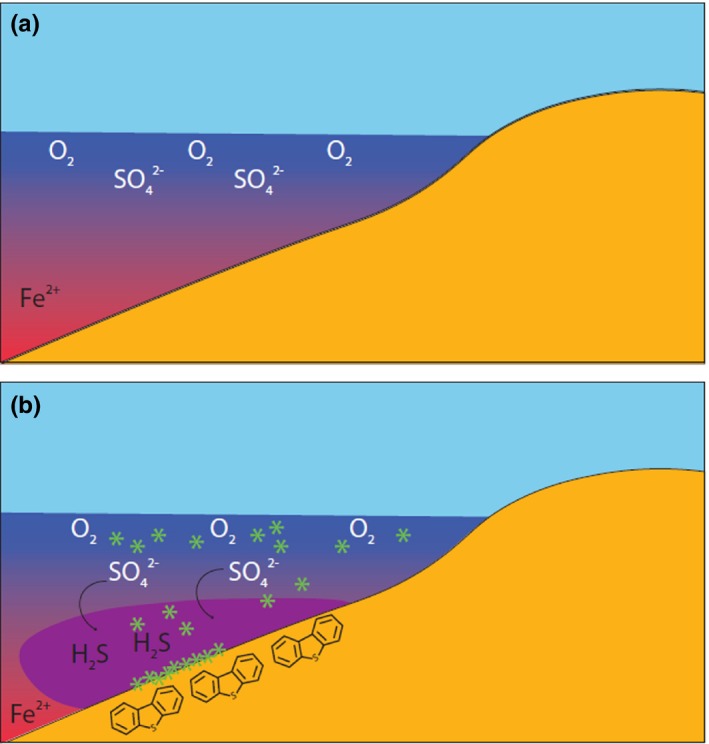
A speculative conceptual model of the chemistry of the Roper Seaway through the (a) upper and lower, and (b) middle Velkerri Formation. Red shading indicates ferruginous waters, purple euxinic waters, and blue the upper oxygenated water column; green indicates cyanobacteria inhabiting the upper water column. Dibenzothiophene molecule shown in (b) to identify higher concentrations in euxinic sediments. Water column geochemistry is based on Cox et al. (2016) and this study [Colour figure can be viewed at wileyonlinelibrary.com]

Complementary inorganic geochemical proxies suggest that the deep water was sub‐oxic to anoxic throughout the Velkerri Formation, with intermittent euxinia occurring only in the Amungee Member (Figures 2 and 9; Cox et al., 2016). Euxinia correlates with increased DBT/P ratios demonstrating enhanced organic matter sulphurisation (Figure 2). Carotenoids are diagenetically altered by sulphurisation which can enhance preservation (Kohnen, Sinninghe Damste, & De Leeuw, 1991; Payzant, Montgomery, & Strausz, 1986; Schaeffer, Reiss, & Albrecht, 1995). The recorded sub‐oxic conditions mean that sub‐oxic degradation of any TMAIs remains a possibility in the Velkerri Formation. Secondary destruction of biolipids in the sediment is also consistent with the generally low concentration of other biomarkers such as cheilanthanes and hopanes in Velkerri sediments when compared to total hydrocarbon concentrations. In summary, the data most strongly suggest that C_40_ carotenoids and associated 2,3,4‐ and 2,3,6‐trimethyl aryl isoprenoids are not detected as green sulphur bacteria (Chlorobiaceae) and purple sulphur bacteria (Chromatiaceae) were either not significant in the Roper Seaway, or removed due to deepwater sub‐oxic conditions and diagenetic removal. This highlights the utility of a combined inorganic and organic geochemical palaeoenvironmental reconstruction.

### 4.6 Microbial ecology of the Velkerri formation

Biomarkers from drillcore Altree 2 describe an environment dominated by bacteria and little input of biomass from archaea and eukaryotes (Figure 9). Evidence for this includes an abundance of mid‐chained and terminally branched monomethyl alkanes that may be derived from the isomerisation of functionalised bacterial *n*‐alkyl and methylalkyl lipids (e.g., Figure 3; Pawlowska et al., 2013) in addition to regular, rearranged and methyl hopanes (Figures 4 and 5) in the upper half of the Velkerri Formation.

Saturated steranes are below detection limits in all Velkerri Formation extracts. Possible explanations for their absence include thermal destruction, taphonomic bias through selective heterotrophic removal of sterols (Pawlowska et al., 2013), or primary absence of the source organisms. Figure 2 demonstrates that the Wyworrie and part of the Amungee Member samples are immature to early mature. At these maturities, steranes are not in the zone of thermal destruction (Peters et al., 2005). Furthermore, since hopanes have a similar thermal stability threshold as steranes and are detectable to 793.37 m, the absence of detectable steranes in the upper, immature zone of the drillcore is clearly not caused by thermal destruction. Pawlowska et al. (2013) suggested that the paucity of steranes in many mid‐Proterozoic shallow‐water environments may be caused by extreme heterotrophic reworking of planktonic eukaryotic organic matter within the upper, oxygen saturated layer of phototrophic microbial mats (“mat‐seal hypothesis”). However, the black organic‐rich siltstones of the Velkerri Formation were clearly deposited in a relatively deep, sub‐storm wave base environment where oxygen‐producing benthic mats cannot thrive. Also unlikely is the selective removal of eukaryotic biomass through extensive recycling within the water column as suggested by Pawlowska et al. (2013). Larger, fast sinking eukaryotic particles would have a shorter residence time in the water column than slow‐sinking bacterial debris and should be preferentially preserved rather than destroyed (Gueneli et al., 2018). Thus, selective and extreme heterotrophic sterol removal is not a likely scenario for the paucity of steranes in the Velkerri Formation, and the most likely explanation is that modern‐day equivalent, sterol‐producing aerobic eukaryotes were not a significant component of the pelagic ecosystem. This is consistent with an absence of steranes in the indigenous Velkerri Formation extracts analysed by Flannery and George (2014) from other locations, suggesting the results from Altree 2 are likely spatially similar through the Roper Seaway, and are consistent with a general lack of steranes in all other mid‐Proterozoic basins where indigenous biomarkers have been recovered (Gueneli et al., 2018).

Based on the current record, indigenous steranes only emerge in the Tonian (1,000–720 Ma). However, Tonian steranes maintain a primitive homologue distribution with a nearly 100% predominance of cholestane (C_27_), and traces of ergostane (C_28_) and the unique sterane cryostane (26‐methylcholestane; Adam, Schaeffer, Paulus, & Brocks, 2017; Brocks et al., 2016). Stigmastane (C_29_) remains undetectable in the Tonian (Hoshino et al., 2017). Sterane diversity and abundance dramatically increase in the Cryogenian (720–635 Ma), in the warm period between the Sturtian and Marinoan Snowball Earth glaciations 659–645 Ma ago, heralding the rise of algae as ecologically important primary producers in the oceans (Brocks et al., 2017). The siltstones of the Velkerri formation were thus laid down several hundred million years before eukaryotes became a notable component of marine ecosystems, and the paucity of steranes, and presence of hopanes, points to phototrophic bacteria as the dominant primary producers in the Roper seaway.

It has been hypothesised that anoxic and nutrient‐limited open waters in the Mesoproterozoic were hostile to the expansion of eukaryotes (Anbar & Knoll, 2002). Eukaryotic microfossils have been identified in shoreline facies of the Roper Group (Corcoran and Jalboi formations) and decrease in species abundance and diversity towards the deeper water Roper Group facies (Javaux et al., 2001).

A transect of bulk nitrogen (δ^15^N_bulk_) isotopes through the Roper Group demonstrated a lateral gradient in nitrate availability with an increase towards shallow nearshore environments (Koehler, Stüeken, Kipp, Buick, & Knoll, 2016). This may help explain the microfossil distributions (Javaux et al., 2001) in the Roper Group with the abundance of microfossils recorded in nearshore environments in the Roper Group being due to the restriction of nutrients rather than taphonomic bias. It is also consistent with the absence of steranes in the deep‐water facies of the Roper Group (this and previous biomarker studies). However, no biomarkers have been reported to date from more oxygenated, fossiliferous shallow‐water facies because these facies are commonly organically too lean to yield hydrocarbons. The search for eukaryotic biomarkers in Mesoproterzoic shallow‐water facies is subject of future research.

In source rocks, the UCM is commonly attributed to either reworking of the organic matter by heterotrophic micro‐organisms either in the water column, or within a microbial mat (e.g., Craig et al. 2013; Li, Cao, Hu, & Luo, 2015). Similar modern environments with microbially derived UCMs include sediments from Lizard Island on the Great Barrier Reef, Australia and St Croix, US Virgin Islands (Reitner et al. 2000), and iron‐rich biofilms in Sweden (Heim, Quéric, Ionescu, Schäfer, & Reitner, 2017). A GCxGC TOFMS study on a UCM extracted from the Mesoproterozoic Xiamaling shale in China proposed two causes of the UCM: microbial activity and enhanced isomerisation. In that study, the presence of 1–3 cyclic paraffins and alkanes with multiple branching positions was interpreted as evidence for intensive isomerisation post‐burial (Li, Cao, Hu, & Luo, 2015; Mißbach et al., 2018). This is also a possibility for the Velkerri Formation as the abundance of cyclic hydrocarbons and MMA and DMA relative to *n*‐alkanes is high in comparison to typical Phanerozoic bitumens. The causes for such enhanced isomerisation in Proterozoic sediments, however, remain obscure.

The very low relative abundances of pristane and phytane, and the absence of both crocetane and the C_20+_ acyclic isoprenoids, demonstrate that archaea were not dominant contributors to the biomass either (Hoffmann, Foster, Powell, & Summons, 1987; Kenig et al., 1995; Peters et al., 2005). By default, bacterial photo‐ and heterotrophs must then have been responsible for the bulk of export production during deposition of the Velkerri Formation.

An increase in DBT/P occurs within the Amungee Member of the Velkerri Formation. Increases in DBT/P are caused by the reaction and incorporation of sulphur species into organic matter during early diagenesis (Hughes et al., 1995; Werne et al., 2008). The absence of the 2,3,4‐ and low concentrations of the 2,3,6‐trimethyl aryl isoprenoids compared with the Barney Creek Formation (Figure 8) suggests that the photic zone of the Roper Seaway may not have been persistently euxinic and may be in direct contrast with the increased DBT/P ratio. Cox et al. (2016) demonstrated Altree 2 contains elevated concentrations of the elements sulphur, molybdenum and vanadium, recording the onset of intermittent deep‐water euxinia, and this is coeval with the increase in DBT/P (Figures 2 and 9; Cox et al., 2016). However, this deep‐water euxinia was intermittent with prevailing conditions characterised by oxic shallow waters and sub‐oxic deep waters; hence, oxic degradation of aromatic carotenoids derivatives is a distinct possibility in the Velkerri Formation including the degradation of aryl isoprenoids and carotenoids and also lower homohopane indices (Table 2). Therefore, the combination of organic and inorganic biomarker proxies leads to an increased understanding of the microbial ecology of the Roper Seaway in the Mesoproterozoic.

### 4.7 Comparison of the Roper Seaway with other Mesoproterozoic environments

Currently, the oldest known indisputably indigenous hydrocarbons have been identified in the *ca*. 1.64 Ga Barney Creek Formation (BCF) of the McArthur Group, McArthur Basin. Hydrocarbons from the BCF include abundant C_40_ aromatic carotenoid derivatives and 2,3,4‐ and 2,3,6‐TMAI assigned to phototrophic purple sulphur bacteria (of the family Chromatiaceae) and green sulphur bacteria (Chlorobiaceae) respectively (Brocks & Schaeffer, 2008; Brocks et al., 2005), β‐carotene presumably derived from cyanobacteria (Lee & Brocks, 2011) and oligoprenyl‐curcumanes that may play a role in the oxidative stress response in bacteria (Brocks, Bosak, & Pearson, 2009). However, as in the Velkerri, diagnostic eukaryotic steranes were not detected (Figure 7 in Brocks et al., 2008). These biomarkers indicate phototrophic bacterial activity in the oxygenated mixed zone and in underlying anoxic waters within the photic zone of the water column. Independent inorganic geochemistry including iron speciation and sulphur isotope systematics supports euxinic conditions in the BCF (Johnston et al., 2008; Shen, Canfield, & Knoll, 2002). These geochemical conditions and biomarker assemblages are broadly comparable to black, finely laminated shales from the *ca*. 1.1 Ga El Mreïti Group in the Taoudeni Basin, Mauritania (Blumenberg et al., 2012; Gueneli et al., 2018). There, the presence of 2,3,4‐ and 2,3,6‐TMAI, in addition to iron speciation, and sulphur isotopes of pyrite and redox‐sensitive trace elements all suggest nearshore PZE conditions (Blumenberg et al., 2012; Gilleaudeau & Kah, 2015; Gueneli et al., 2018; Kah, Bartley, & Teal, 2012). Moreover, the nitrogen isotopic composition of porphyrins from the El Mreïti Group confirms that primary productivity was dominated by bacteria (Gueneli et al., 2018), and there is no fossil (Beghin, Guilbaud, et al., 2017; Beghin, Storme, et al., 2017) or biomarker evidence for the activity of algae (Blumenberg et al., 2012; Gueneli et al., 2018). Thus, the combination of these studies, in addition to trace metal redox work in other locations, suggests environmental heterogeneity in the Mesoproterozoic (e.g., Canfield, 1998; Lyons, Anbar, Severmann, Scott, & Gill, 2009).

Earlier studies suggested that euxinia was a defining feature of most deep Mesoproterozoic basins and the open ocean (see reviews by Lyons, Reinhard, & Planavsky, 2014; Canfield, 2014). However, as more data accumulate it becomes clear that euxinia was not widespread (Cox et al., 2016; Planavsky et al., 2011; Poulton, Fralick, & Canfield, 2010; Sperling et al., 2014). Poulton et al. (2010) demonstrated that euxinia was spatially restricted and formed wedges proximal to the continental shelf. Furthermore, there are locations where ferruginous conditions appear to dominate the entire system (Cumming, Poulton, Rooney, & Selby, 2013; Planavsky et al., 2011). The *ca*. 1.44 Ga Hongshuizhang Formation and the 1.38 Ga Xiamaling Formation, both in northern China, are inferred to have been deposited under anoxic, but not sulphidic conditions (Luo, George, Xu, & Zhong, 2016; Luo, Hallmann, Xie, Ruan, & Summons, 2015), with the possibility of oxygenation for example in unit three of the Xiamaling Formation (Wang et al., 2018; Zhang et al., 2016). In those studies, water column chemistry was reconstructed using redox‐sensitive trace metals and biomarkers, including the absence of aryl isoprenoids, reducing the probability of photic zone euxinia (Luo et al., 2015, 2016).

Furthermore, the *ca*. 1.1 Ga lacustrine Nonesuch Formation, USA, is an additional example of a ferruginous water body based on extensive Fe‐C‐S systematics (Cumming et al., 2013). Aryl isoprenoids are also below detection limits in the Nonesuch Formation (Imbus, Engel, Elmore, & Zumberge, 1988; Pratt et al., 1991), strengthening the argument against PZE, although the absence of these hydrocarbons may also be due to other factors including thermal destruction. In addition, a persistently oxic water column in the 1.4 Ga Kaltasy Formation in the Ural Mountains, central Russia has been identified based on iron speciation (Sperling et al., 2014).

The biomarker assemblage of the Velkerri Formation is similar to those reported for the Hongshuizhang, Xiamaling and Nonesuch formations, with biomarkers for PZE being absent or in low concentrations suggestive of transient anoxia and/or intermittent flux of oxygen to bottom waters, which contrasts with the permanently anoxic Barney Creek Formation in the McArthur Basin. Differences between the sedimentary basins in northern China and the Velkerri Formation of the McArthur Basin have recently been discussed by Luo et al. (2016). Pr and Ph are in relatively higher concentration in the Hongshuizhang, Nonesuch and Xiamaling formations compared with the Velkerri Formation. Additionally, the Xiamaling contains trace concentrations of diahopanes and increased concentration of dibenzofuran interpreted to be biomarkers for aerobic diagenesis (Wang et al., 2018). Luo et al. (2016) suggest that the microbial input between these three formations could be varied; however, it is also likely that variations in hydrocarbon distributions are influenced by processes in the water column and bottom sediments including clay‐catalysed isomerisation reactions (e.g., Alexander, Kagi, & Larcher, 1984), the amount of sulphurisation (Schaeffer et al., 1995) and oxygen exposure times (e.g., Charrie‐Duhaut, Lemoine, Adam, Connan, & Albrecht, 2000).

In summary, the ecology and chemistry of mid‐Proterozoic marine basins were not all the same. Globally, mid‐Proterozoic sedimentary basins reveal a more diverse water column chemistry and preserve a wider range of organic compounds than assumed a decade ago Inevitably, the identification and analysis of a larger sample set of suitable sedimentary successions for biomarkers, microfossils and inorganic geochemistry of different depositional environments, in particular nearshore and oxygenated sedimentary basins, will lead to a greater insight into the relationship between early complex life, biogeochemical cycles and marine and atmospheric redox in the Mesoproterozoic.

## 5 CONCLUSIONS

Indigenous hydrocarbon biomarkers extracted from siltstones in the Velkerri Formation of the McArthur Basin from drillcore Altree 2 have been used to determine the microbial ecology of the *ca*. 1.38 Ga Roper Seaway. The saturated biomarker assemblage includes regular, rearranged and methyl hopanes suggesting that the organic matter was dominantly produced and subsequently reworked by bacteria. Steranes were below detection limits in all extracts analysed despite eukaryotic microfossils identified in nearshore sediments of the Roper Group, suggesting that eukaryotes, while present in the Roper Seaway, were ecologically restricted and contributed little to the net biomass of the deep‐water facies. This work confirms the similar findings of Flannery and George (2014) who analysed three samples from other Velkerri Formation cores suggesting the results from Altree 2 are likely spatially similar through the Roper Seaway.

Elevated dibenzothiophene/phenanthrene (DBT/P) from the Wyworrie to Amungee Members of the Velkerri Formation broadly correlates with increasing sulphide concentrations in the deep‐water column and TOC abundance. The elevated sulphide levels would have been subsequently incorporated into the organic matter, broadly consistent with the onset of euxinia (based on coeval enrichments in TOC, Mo, V and U; see Cox et al., 2016 at similar depths in the drillcore). The 2,3,4‐ and 2,3,6‐trimethyl aryl isoprenoids (TMAI) were absent or in very low concentration in the Velkerri Formation. The low abundance is primary and not caused by thermal destruction. Instead, TMAI abundances were either affected by degradation of carotenoids during intermittent oxygen exposure at the sediment–water interface and/or the water column was rarely euxinic in the photic zone and likely only transiently euxinic at depth (>100 m). A comparison of this work with recently published biomarker and trace elemental studies from other mid‐Proterozoic basins demonstrates that microbial environments, water column geochemistry and basin redox were heterogeneous.
